# A Highly Pipelined and Highly Parallel VLSI Architecture of CABAC Encoder for UHDTV Applications

**DOI:** 10.3390/s23094293

**Published:** 2023-04-26

**Authors:** Chen Fu, Heming Sun, Zhiqiang Zhang, Jinjia Zhou

**Affiliations:** 1 Graduate School of Science and Engineering, Hosei University, Tokyo 184-8584, Japan; 2 Waseda Research Institute for Science and Engineering, Waseda University, Tokyo 169-8050, Japan

**Keywords:** high efficiency video coding (HEVC), entropy coding, context adaptive binary arithmetic coding (CABAC), video coding, hardware design

## Abstract

Recently, specifically designed video codecs have been preferred due to the expansion of video data in Internet of Things (IoT) devices. Context Adaptive Binary Arithmetic Coding (CABAC) is the entropy coding module widely used in recent video coding standards such as HEVC/H.265 and VVC/H.266. CABAC is a well known throughput bottleneck due to its strong data dependencies. Because the required context model of the current bin often depends on the results of the previous bin, the context model cannot be prefetched early enough and then results in pipeline stalls. To solve this problem, we propose a prediction-based context model prefetching strategy, effectively eliminating the clock consumption of the contextual model for accessing data in memory. Moreover, we offer multi-result context model update (MCMU) to reduce the critical path delay of context model updates in multi-bin/clock architecture. Furthermore, we apply pre-range update and pre-renormalize techniques to reduce the multiplex BAE’s route delay due to the incomplete reliance on the encoding process. Moreover, to further speed up the processing, we propose to process four regular and several bypass bins in parallel with a variable bypass bin incorporation (VBBI) technique. Finally, a quad-loop cache is developed to improve the compatibility of data interactions between the entropy encoder and other video encoder modules. As a result, the pipeline architecture based on the context model prefetching strategy can remove up to 45.66% of the coding time due to stalls of the regular bin, and the parallel architecture can also save 29.25% of the coding time due to model update on average under the condition that the Quantization Parameter (QP) is equal to 22. At the same time, the throughput of our proposed parallel architecture can reach 2191 Mbin/s, which is sufficient to meet the requirements of 8 K Ultra High Definition Television (UHDTV). Additionally, the hardware efficiency (Mbins/s per k gates) of the proposed architecture is higher than that of existing advanced pipeline and parallel architectures.

## 1. Introduction

The creation of intelligent sensor nodes that enable intelligent processing for Internet of Things (IoT) surveillance, remote sensing, and smart city applications is gaining more and more attention [1]. In this, video data is crucial, and specifically designed video codecs have been preferred in recent years [2]. With a focus on reducing the data burden and improving the video quality [3], video coding and processing techniques performed in low-cost implementations and higher compression efficiency will cope with the design requirements of sensor nodes. The Joint Collaborative Team on Video Coding (JCT-VC) published the High Efficiency Video Coding (HEVC) standard in 2013 [4]. With a more flexible block division structure, a more precise coding mode, and some cutting-edge coding tools, HEVC is the widely used worldwide video coding standard [5].

The HEVC standard’s coding structure primarily comprises Prediction, Estimation, Motion compensation, Quantization and Transform, and Entropy coding. The video pixel value, which is broken down into two chrominance channels and one brightness channel, serves as the input for this coding system. The image is chunked into coding tree units (CTUs), which support a range of sizes [6]. Intra and inter frame prediction is first carried out to encode this CTU video block [7]. The rate-distortion cost is then assessed using various prediction modes, block size, and distortion degree, and the block segmentation method and the prediction mode of this CTU are obtained [8]. To produce the converted coefficient known as “residual”, the original pixel data must be subtracted from the predicted data in order to obtain the difference [9]. The difference is then further transformed and quantized [10], and some high-frequency components are removed. To create the code stream, the projected data and the residuals will be entropy coded.

Entropy coding is a coding class that performs lossless coding based on the information entropy principle. Contrary to the Context-based Adaptive Variable Length Coding (CAVLC) and CABAC hybrid coding approach employed by the previous generation video coding standard AVC/H.264 [11], HEVC/H.265 only uses the CABAC entropy coding method.

A high-performance entropy encoder remains one of the hardware implementations’ constraints for entropy coding in video coding. The amount of data that must be processed via entropy coding in HEVC is also significantly increased to handle more complex Rate Distortion Optimization (RDO) operations and Syntax Elements (SEs), which places more demands on hardware implementation. Parallel processing is challenging to implement because of the stringent data reliance of the binary arithmetic coding employed in CABAC, as well as the complexity of the arithmetic coding procedures, which might make it challenging to increase the primary frequency [12,13]. Ding et al. [14] proposed an optimized CABAC “Producer–Consumer” architecture through data flow modeling to achieve high throughput and low resource consumption. Wahiba et al. [15] proposed the processing of 1 to 5 bypass bins at the same by duplicating the number of bypass encoding engine (BEE) blocks for improving the throughput to be transmitted or stored. Ramos et al. [16] presented a novel scheme for multiple bypass bin processing, named multiple bypass bin scheme (MBBS), and the proposed method application into a baseline binary arithmetic encoding (BAE) architecture, showing an increasing bin per cycle throughput. Li et al. [17] considered the bypass mode encoding process in the CABAC and tried to merge bypass bins, and implemented one clock to encode six bins in bypass encoding mode to improve throughput. Zhou et al. [18] proposed and implemented in hardware a series of throughput improvement techniques: pre-normalization, Hybrid Path Coverage, Lookahead rLPS, bypass bin splitting and State Dual Transition, and by combining all these optimizations, overall CABAC performance improved by leaps and bounds.

The throughput rates of the SE generation and processing module and the BAE module are essential because they are two modules that both supply and process data. Consequently, we must address the latency that the complex data preparation required by the higher-level modules results in. Wahiba et al. [19] propose a new Register Transfer Level (RTL) architecture of HEVC CABAC encoder, where all SEs transmitted for 4 × 4 sub-blocks are studied and implemented. Saggiorato et al. [20] propose a novel efficient multi-core architectural approach, named Multiple Residual Syntax Element Treatment (MRSET), to meet the requirements of these recent CABAC designs. Tran et al. [21] and Nagaraju et al. [22] propose efficient hardware implementations of binarization for CABAC that focus on low area cost and power consumption while providing enough bins for high-throughput CABAC.

There is a problem that they need to address specifically, even though the current work considerably increases the throughput of CABAC encoders. When encoding successive bins of the same context model in BAE, the pipeline or parallel architecture of CABAC periodically stall, decreasing the coding efficiency. This paper aims to improve the performance further and enhance the compatibility of the entropy coding module, which is used to ensure the overall video coding architecture and the continuous and stable operation of this entropy coding encoder. This study builds on our earlier work by offering several fresh architectural modifications to enhance the critical path delay and the number of bins provided every clock cycle, dramatically increasing the overall throughput. Below is a summary of this paper’s significant contributions.

1. We examine the challenges and bottlenecks in pipelined or parallel implementations brought on by arithmetic coding’s back-and-forth dependency on coding states. We propose to use pre-range update and pre-renormalize technique to reduce the multiplex BAE route delay due to the incomplete reliance of the encoding process.
2. We propose the variable bypass bin incorporation (VBBI) technique, which allows an extra two bypass coding bins to be processed in the same clock cycle in a quad parallel architecture, significantly improving the throughput of BAE in a parallel architecture.
3. When the context model cannot be prefetched early enough, the pipeline will stall since the context model needed for the current bin typically depends on the outcome of the previous bin. We provide a prediction-based context model prefetching strategy to address this issue. Additionally, the Multi-result Context Model Update (MCMU) architecture is proposed, the critical path for state transitions is shortened by the context model update of the meticulously optimized parallel architecture.
4. Based on the HEVC video coding standard, a highly compatible hardware architecture for entropy encoding is provided. The whole entropy encoding architecture is pipelined, and the data interaction between binarization and BAE is cached using parallel-in-parallel-out (PIPO) to improve the stability of the entropy encoder. It also develops a quad-loop cache architecture to improve compatibility for data interaction between the entropy encoder and other video encoder modules.

## 2. Analysis of CABAC

### 2.1. CABAC’s Process

**Figure 1:**
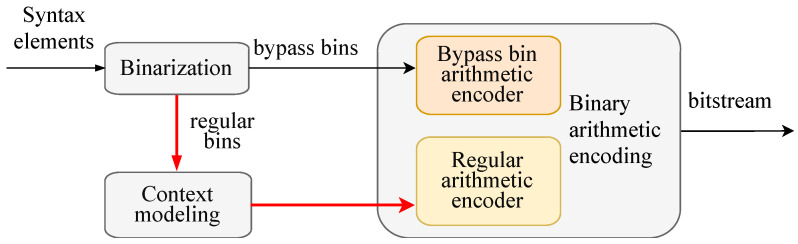
Key components of CABAC.

As depicted in Figure 1, CABAC comprises three key modules: binarization, context modeling, and binary arithmetic coding [23]. The video prediction data, reference data, etc., are parsed into the appropriate SEs in the entropy coding process. These SEs include prediction patterns, block segmentation flag, etc. After binarization, the values of the non-binarized SEs are mapped into a series of a variable number of binary symbols [22]. Each binary symbol is referred to as a bin. The critical information of the video sequence is represented by the syntax elements, which aim to represent the video with the least amount of data possible while allowing for the reconstruction of the video sequence at the decoding stage.

The binary symbol bin is the data that can be processed directly by the arithmetic coding module. Arithmetic coding is primarily split into Regular Coding and Bypass Coding, with various SEs accessing distinct selection criteria for each. Among them, the context modeling part will supply the context probability model of the associated bin based on the context data from the SEs for the regular coding bin.

The HEVC standard defines several binarization methods for entropy coding: Fix-Length (FL) coding, Truncated Rice (TR) coding, K-order exponential Golomb coding, etc. The above binarization methods are the most critical for syntax elements in HEVC, except for very few syntax elements with their own specific binarization methods. This is mainly influenced by the numerical characteristics of different SE values and is related to the context model selection methods corresponding to other SEs. In addition, although the binarization method of SEs is specified directly by the standard, the quantization parameters cMax and RiceParam often depend on the specific encoding situation. For example, the cMax parameter of the merge_idx index is determined by the number of merge mode candidates.

The probability of encoded blocks and encoded SEs is reflected in the context model in entropy coding. The core of context modeling is to estimate the distribution probability of the currently encoded SEs and enhance coding efficiency by using video data’s spatial and temporal correlation. The accuracy of context modeling, which holds a key place in the entropy coding standard, significantly affects the coding effect. For the standard coding model, the coding procedure for each bin includes the corresponding context model. To adaptively make adjustments to diverse videos, these context model need to be updated in real time.

**Figure 2:**
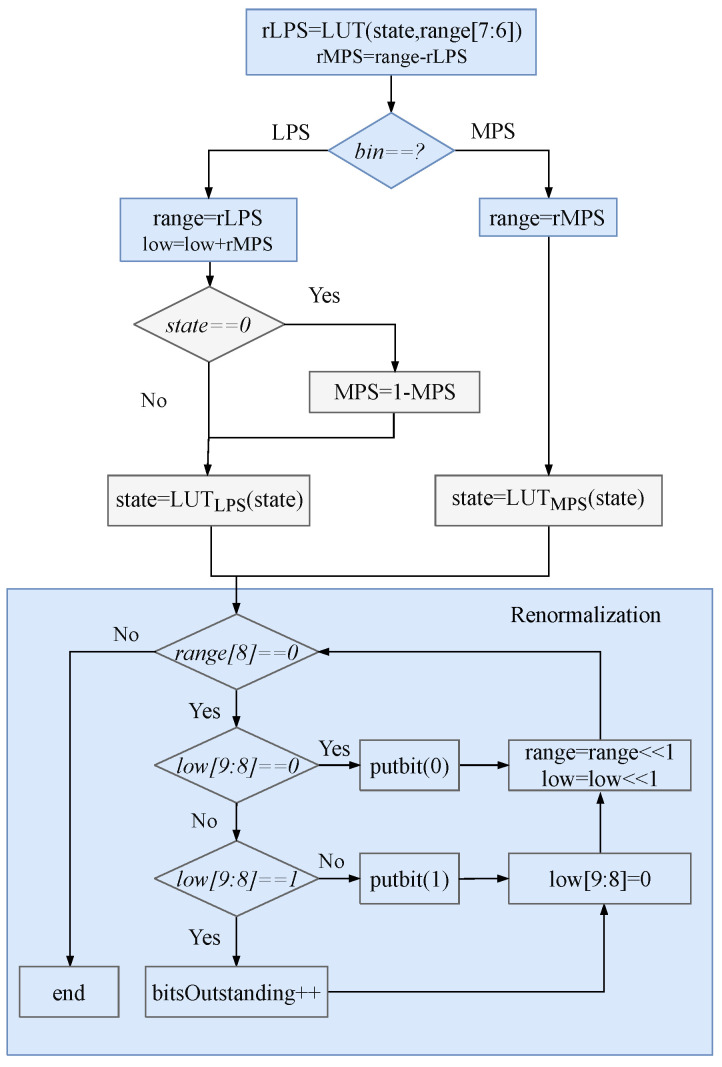
Flowchart of BAE. The gray section can be pre-executed before the blue section.

Although the arithmetic coding specified by the HEVC standard is conceptually comparable to the joint finite-precision binary arithmetic coding, numerous modifications have been made to the implementation techniques to reduce the complexity of the operations. The More Probable Symbol (MPS) and Less Probable Symbol (LPS) definitions of the encoding’s binary symbols denote the symbols having a big and small probability of occurrence, respectively. The binary arithmetic encoding inputs are the bin to be encoded and its accompanying contextual model. Figure 2 depicts the encoding procedure, primarily separated into the MPS and LPS bin types. Although the two flow lines are different, they include stages like renormalization, calculating rLPS and updating the context.

### 2.2. Bottleneck Analysis

**Figure 3:**
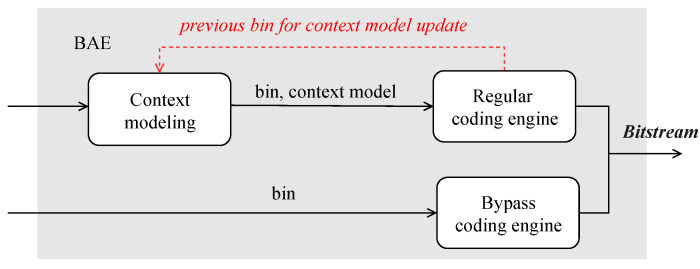
Each time a bin is encoded in the regular coding engine, the context model must be changed and saved back into the Context Modeling.

The pipeline architecture is one successful approach to increasing the throughput of BAE hardware, and the multi-channel parallel architecture is another. BAE in HEVC suffers from a huge area of memory due to lots of context models [24,25], so Static Randomaccess Memory (SRAM) is used instead of registers. However, a particular case in the implementation causes the pipeline architecture to stall. As shown in Figure 3, when the current bin coding is complete, the context model of the same bin must be restored for the next bin at the next clock cycle. Updating the context model requires one clock, and reading or writing the context model from RAM also consumes one clock, so subsequent bins cannot read the updated context model from the adjacent clock from the context model RAM that has not yet been written. Therefore, it is necessary to suggest a CABAC hardware design that can implement a parallel or pipelined CABAC without stalling.

For multiplexed parallel context model update architectures, the resulting path delay corresponds to many levels of multiplexers, which will dominate the critical path of CABAC. Thus, bottlenecks have emerged in determining how to improve the efficiency of the pipeline/parallel structure, and use less hardware to achieve better throughput CABAC designs.

For a variety of data, other video encoder modules communicate with the entropy encoding. Numerous data will be combined in the entropy coding. The entropy coding may occasionally fail to finish digesting the input data in a timely manner, resulting in the loss of the input data since its coding efficiency differs from that of the other modules of the encoder. The residual coefficient data are the largest class of data among the coded data required for entropy coding. It also becomes challenging to balance the data supply of the reconstruction module with the value of the entropy coding and how to store these data more effectively.

## 3. Proposed CABAC Prediction-Based Context Model Prefetching Strategy

### 3.1. Prediction-Based Context Model Prefetching

One of the features of CABAC is that each time a regular encoding is performed, the probabilistic model of the current encoded bin needs to be updated. The context modeling needs to transmit the same throughput to support the BAE with multiple bins constructed above.

However, pipeline or parallel implementation is complex when faced with some exceptional cases. When there are successive bins with the same context model, since one clock is required to update the context model and both reading and writing of RAM data also occupy one clock, the latter bin cannot be read from the context model memory CM_RAM in the adjacent clock cycle that has not yet been written to the updated context model. To cope with the phenomenon of pipeline stall, this paper proposes a context model prefetching strategy and optimizes it for the multi-bin case, aiming to achieve a stall-free pipeline and low resource and high master frequency.

**Figure 4:**
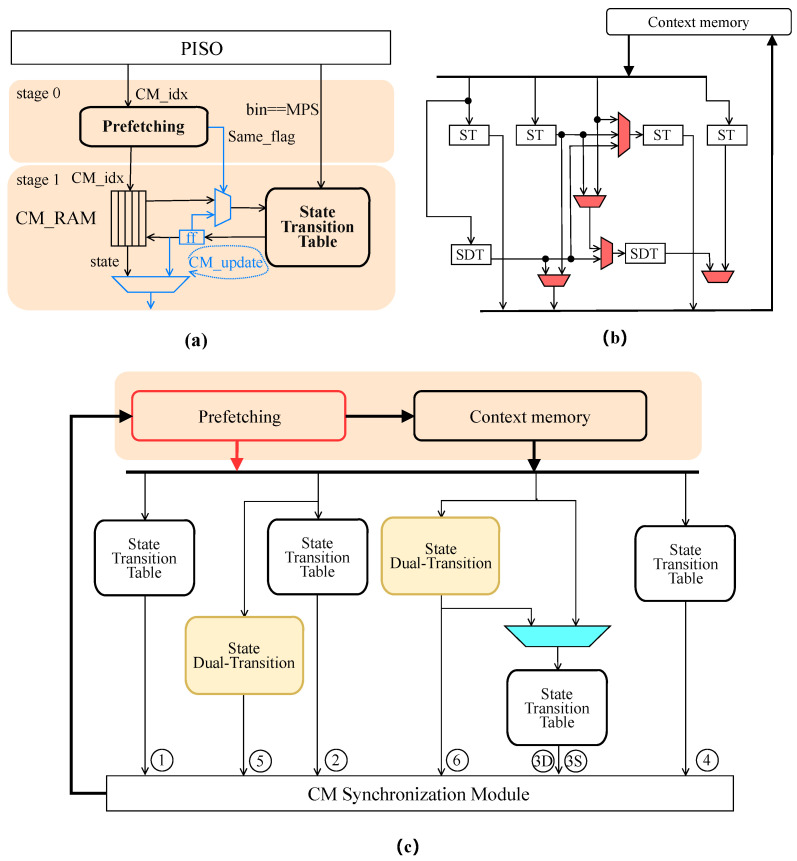
Proposed SDT-based context updating architecture. ST and SDT are 128-1 and 256-1 LUTs, respectively. (**a**) Proposed architecture for updating single-way pipeline context model based on prefetching. (**b**) Architecture proposed by [18] with additional state dual-transition (SDT) LUTs. (**c**) Proposed MCMU architecture. The values from 1 to 6 denote the possible outputs.

The context modeling architecture of the pipeline BAE in this paper is shown in Figure 4a. Because only one bin is processed per cycle, the design of this paper uses Parallel In Serial Out (PISO) as the input module for context model update. The PISO module outputs data for one bin at each clock cycle. At the same time, the context model needs to be obtained from CM_RAM by index. Prefetching will save the relevant data and predict the next incoming bin to be the same context model as the current bin. Finally, the predicted bin values and other data are transferred to the next stage. Since RAM reading and writing consume one clock cycle, if consecutive bins utilize the same context model, the post-context model cannot access the data written after the pre-update of the adjacent clock cycle.

When the index of the current clock cycle input to CM_RAM is the same as the previous clock cycle, the context model is directly communicated to stage 1 of the pipeline through the Same_flag, and the context model is directly passed through the internal pipeline. The CM update module receives the updated model in the previous clock cycle instead of using CM RAM. Thus, regardless of whether the context model of the next bin is the same as the current consistent one, the correct data can be output promptly. The context model that needs to be updated is found in the state transition table and then saved in CM_RAM for real-time updates. This is a prefetching strategy proposed in this paper to solve this case, implemented by caching the model’s index.

As depicted in Figure 4b, ref. [18] designed an architecture for context model update in parallel architecture. However, the critical path must be further optimized to prevent the critical path delay from exceeding BAE. To address this issue, we propose the Multi-result Context Model Update (MCMU) architecture in this paper.

Ref. [18] had to make the probabilistic state update satisfy all bin cases; many multiplexers are placed between state transition (ST) and state dual-transition (SDT), which is considered to be simplified in this paper, and the new architecture is shown in Figure 4c. The architecture utilized in this work features one clock cycle for encoding up to four bins, where the type of bin specified in Table 1 denotes the interrelationship of the context models of these four bins. When the bin type is the same, as indicated by the same context model, it yields a total of only seven cases. For instance, if the bin type is ABBD, this means that the middle two bins utilize the same context model. The proposed architecture provides six results per clock cycle. Among them, result 3 contains two cases that must be arbitrated by prefetching. Therefore, this architecture can obtain all the results of the context model update by only one multiplexer, at the cost of dropping the encoding of the last bin if all the four bins are of the same context model, i.e., only three bins are encoded in parallel in this clock cycle.

Suppose there are bins with the same context model in the next clock cycle. In that case, the context model needs to transfer to the BAE module first through the cache in time to avoid the untimely transmission of the context model due to the read and write time of the memory.

### 3.2. Proposed Pre-Range Update and Pre-Renormalize BAE Architecture

The context model update, computation of *range*, calculation of *low*, and the renormalization procedure are all carried out in a cascading manner, as is already noted, making the entire coding process feasible for pipelining activities.

A valuable fact for designing a single-way arithmetic coding pipeline is that the *low* of the current coding interval depends on *range* unidirectionally. In contrast, *range* does not depend on *low*. Therefore, in the design of the arithmetic coding pipeline, *range* and *low* can be calculated separately, and *range* is calculated first before *low* to shorten the critical path of the pipeline.

According to Figure 2, the *range* computation includes rLPS lookup, interval recursion, renormalization lookup and shift; *range* update depends on rLPS, and rLPS depends on the current *range* and context model state. Therefore, compared with the *low* update and bitstream output part, the computation of *range* is the most complex part of the pipeline, and the loop algorithm generated by renormalization becomes a bottleneck for the hardware architecture.

However, one renormalization can only double the encoding interval of less than 256, so it is often necessary to perform multiple renormalizations due to the small *range*, which makes it difficult to pipeline and affects the encoding efficiency. Since only the shifting of *range* and *low* and the counting of *bitOutstanding* are performed in the renormalization process, multiple renormalizations can be completed in one operation. The times of renormalizations differ when the bin is MPS and LPS. When the bin is MPS, if *range* is less than 256, then renormalization is performed once; otherwise, renormalization is skipped. Renormalization is required when the bin is LPS, as illustrated in Table 2; renorm count is retrieved from the renormTab table. The lookup table uses the higher five bits of the rLPS as an index for the times of renormalizations.

**Figure 5:**
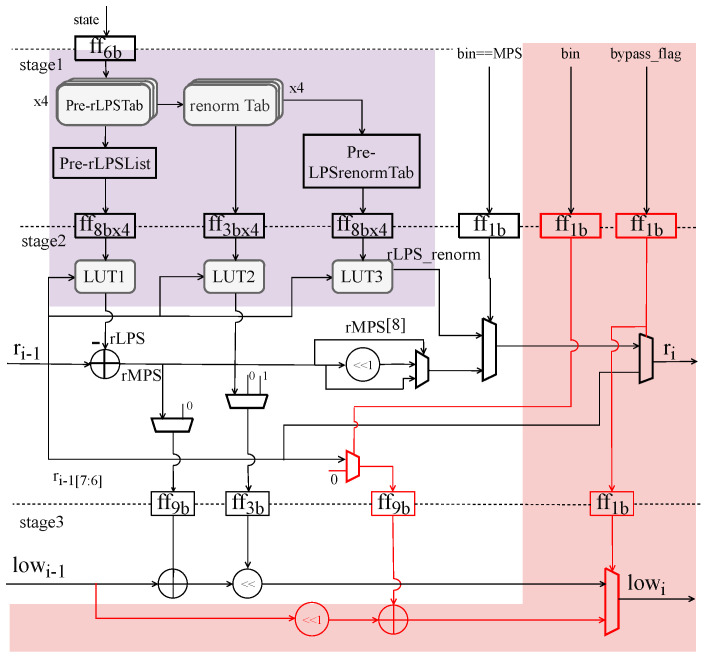
Diagram of pipelined single-bin BAE architecture. The purple part is the proposed pre-renormalize technique. The red part is the single-way hardware architecture which is bypass bin compatible.

Therefore, this paper proposes pre-range update and pre-renormalize, as shown in the purple area of Figure 5. In the first stage, in addition to completing the pre-computation rLPS, the pre-lookup table and storage structure of the renormalization count renorm_count are added. The *range* update is split into two levels of pipeline. Renorm_count is obtained from rLPS by indexing the table. The renormTab table size is 1 × 32, so in the context of the first stage pipeline to obtain four candidate rLPSs, we can also look up the table to obtain the renormalization number renorm_count which corresponds to the candidate rLPS, and shift to obtain the corresponding renormalization interval rLPS_renorm with four candidate values. So the renormalization count lookup table of rLPS can also be split into sub-operations carried out in the first stage pipeline. In contrast, the shift operation in renormalization is completely placed in the first stage pipeline.

The four candidate renormalization values obtained after pre-renormalize will also be used as indexes by *range* [7:6] at the beginning of the second stage pipeline to determine the final rLPS renormalization interval. For the renormalization of MPS, the above pre-normalization method cannot be used because it depends entirely on the coding interval *range* of the previous encoding. In the second stage of the pipeline, the highest bit of rMPS is used as the judgment condition to determine whether to perform rMPS renormalization. The final *range* is selected between rLPS_renorm and rMPS_renorm according to whether the bin is MPS or not.

### 3.3. Area-Efficient BAE Pipeline Architecture with Compatibility

In the entropy coding of HEVC, the bins of regular and bypass coding are sequentially arranged. If hardware is designed separately for both, it can achieve very high coding speed under certain circumstances, especially for bypass bins. The bypass bin splitting (BPBS) described by [18], which increases the throughput of 1 clock cycle, has several implementation limitations. Still, at the cost of memory, resources to store intermediate results and additional bin sequences merge to integrate. For up to five pathways of [18] alone, 32 combinations of bin cases are included. It is also required to allocate all the results in one clock cycle before the update of *low* can be performed. If more multiple bypass bins are attempted, more cases need to be processed, which will be an extremely complex process that will consume a large hardware area and may become a new critical path. So this paper proposes area-efficient BAE pipeline architecture with compatibility.

The coding state is calculated differently for different coding modes, as shown in Table 3. The bypass flag of the current bin is stored in the second stage of the pipeline to select the encoding state computed in different encoding modes. The bypass encoding *range* remains unchanged, with only *low* changes. Our work integrates the bypass and regular encoding in one hardware architecture. The update of *range* is currently the critical path, so combining the update of bypass coding *low* into the pipeline does not cause the frequency to decrease.

The third stage of the pipeline architecture designed in this paper is the update calculation of *low*. The number of renormalizations when the bin is LPS is obtained via renormTab and LUT2 jointly checking the table. If the bin is MPS and rMPS is greater than or equal to 256, the renormalization is skipped, and if the bin is MPS but rMPS is less than 256, the renormalization is carried out once. The bypass coding only updates *low*, and its coding process is shown in the red part in Figure 5. Compared to the design presented in the previous work, the current architecture can accomplish stable and continuous coding with a lower circuit area without extending the critical path.

### 3.4. Multi-Bin Parallel Architecture Based on Variable Bypass Bin Incorporation

A pipeline or parallel architecture are two efficient ways to increase the throughput rate of arithmetic coding technology. However, the dependency on the arithmetic encoding states makes the issue of long-timing routes in the pipeline structure even worse. This work presents a pipeline architecture for the arithmetic encoder and a multi-path parallel architecture with a single pipelined arithmetic encoder on each lane.

**Figure 6:**
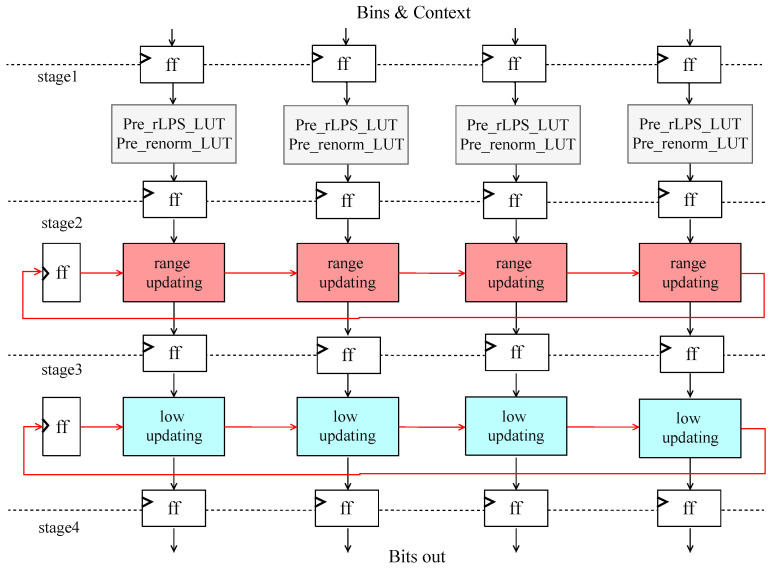
Pipelined multibin BAE architecture.

In the four-way parallel structure shown in Figure 6, the context model updates are precomputed upfront. The *range* and *low* computed by the first encoder are used as the state input for the second channel, and so on for multiple channels of state updates. In particular, the encoding state of the last encoder will be saved in a register as the starting state data for the next set of four-way bin encoding.

In the first stage of the pipeline, in addition to the pre-rLPScalculation and pre-renormalize, a pre-lookup table and storage structure for the renormalization count are added. The renormalization count candidates are stored in registers and will determine the final value in the second-stage pipeline, which will participate in the renormalization calculation of *low*. In a basic four-bin BAE, either a regular or a bypass bin must be encoded sequentially. As we can see through the previous section, the update phase of *range* in a single-path pipeline architecture becomes the critical path, while the update of *low* is more straightforward.

**Figure 7:**
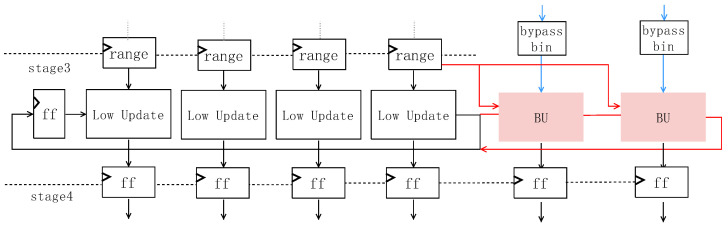
Variable Bypass Bin Integration (VBBI) architecture. It can encode up to 6 bins in 1 clock, including at least two bypass bins.

So this paper proposes the Variable Bypass Bin Incorporation (VBBI) architecture, as shown in Figure 7. By taking advantage of the feature that the bypass bin does not change the context model and *range*, each time four bins are encoded, if immediately followed by one or two bypass bins, these two bins are added to the current bin sequence to achieve the maximum throughput rate of six bins encoded in at most one clock cycle. Even if the update of *low* increases to six bins at the same time, the critical path does not exceed the update process of the *range* of four bins, so there is no impairment in the main frequency performance, and the throughput of the parallel architecture can be effectively increased. Compared with the bypass bin separation architecture used in [18], every single path in this paper can be adapted to bypass coded bins, which not only saves the RAM used to store intermediate variables but also removes the hardware area generated by using bin sequence merge, and the average throughput rate can be achieved very close.

## 4. Overall System

### 4.1. Quad-Loop Cache Input

**Figure 8:**
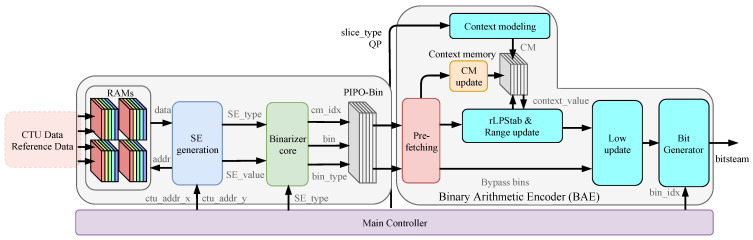
Overall framework of a highly compatible VLSI architecture for H.265/HEVC CABAC encoder for UHD TV applications.

Entropy coding of HEVC is a module that performs statistically based lossless data compression of the results generated by other modules, so it is related to each module in video coding. The coding framework of the entropy coding module is shown in Figure 8. When the entropy coding module obtains all the SEs and residual coefficients, it needs to pre-process the syntax elements and residual coefficients at each level, which includes calculating the values of syntax elements to be coded, the context model index, and the coding method. After the SEs are generated, they will enter the binarization core and input the binarized bin into the PIPO memory. Then the prefetching module will input three to six bins per clock cycle into the BAE, which the bit generator will finally integrate into the bitstream output.

**Figure 9:**
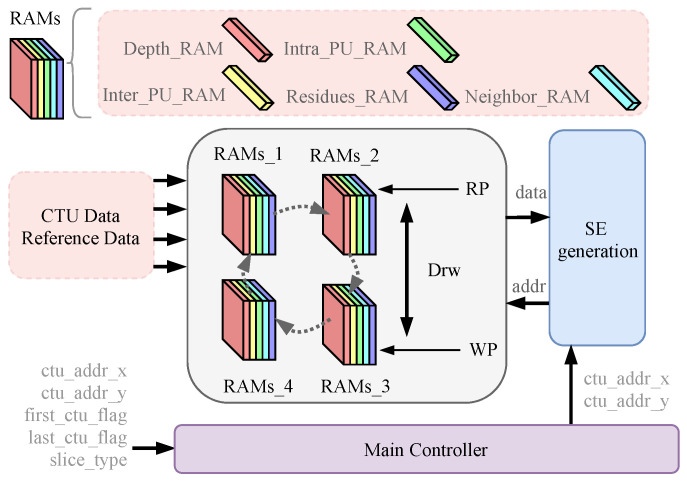
Quad-loop cache architecture.

The data in the entire CTU generated in the video encoding process are passed to the entropy coding module; these data are diverse and need to be considered for hardware architecture to match the timing of transmission. However, the data processing speed of other modules and this module’s throughput will differ. Under the condition that the whole video coding is pipelined architecture, the data input structure, as shown in Figure 9, is used to enhance the compatibility of entropy coding. The quad-loop cache architecture is different from the First-Input-First-Output (FIFO) memory in that it completes the FIFO function for each group of RAMs, and the data in the RAM block can be read out in disorder, which is suitable for the data reading requirement of the entropy coding module. If Drw is 4, Write Pointer (WP) is one turn ahead of Read Pointer (RP) and points to the same RAM as RP. If the pipeline continues to run, it will lead to data loss and coding errors. Therefore, when Drw is equal to 4, the rest of the video encoding process needs to be paused to ensure that the coding is absolutely correct.

The input data include Depth_RAM (containing information on CU depth, TU depth, and PU mode), Intra_PU_RAM (luminance and chrominance direction), Inter_PU_RAM (information related to merge and amvp), Neighbor_RAM (information related to the top side and left side CTU), Residues_RAM (residual data), etc. The data to be entropy coded are cyclically cached through four RAMs. Each group of RAMs keeps all the data of one CTU, effectively reducing the dependency between video coding modules.

### 4.2. Binarization Architecture

The binarization schemes used for most of the SEs in HEVC are Truncated Unary (TU), Truncated Rice (TR), Kth-order ExpGolomb (EGK), and Fixed-Length (FL) codes. The rest of the SEs use their corresponding custom binarization schemes, which will include some compound encoding [26].

Since the binarization is carried out separately for each SE and is not the bottleneck of the whole architecture, as long as the average throughput of the part is higher than the average throughput of the BAE, in any case, the entire architecture can be satisfied with smooth and efficient operation.

**Figure 10:**
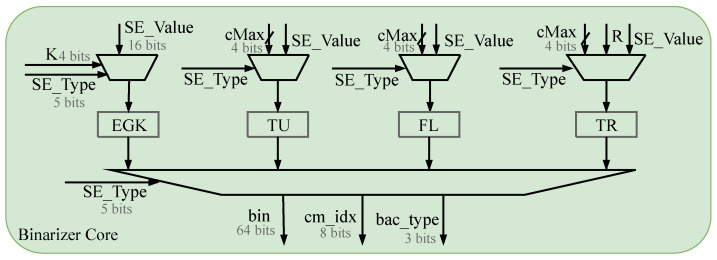
Proposed single-core binarization architecture.

The architecture of the single-core binarization module is shown in Figure 10. The input is SE encoding type value, which is encoded according to the respective encoding rules. The output of the completed encoding are the bin value, the context model index, and the encoding type [27].

**Figure 11:**
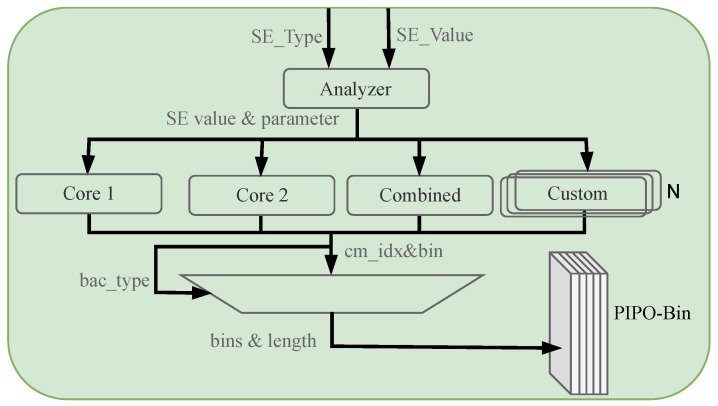
Proposed parallel binarization architecture. It can implement binarization of multiple syntax elements in 1 clock.

This design uses a parallel three-stage binarization scheme to meet the goal of smooth and efficient binarization, as shown in Figure 11. The first stage is responsible for inputting and sorting the syntax element values SE_Value and encoding types SE_Type that need to be binarized in order and then transferring them to the following encoding stage [28]. The second stage is responsible for binary encoding. It consists of two single-core binary modules, one combined module, and one custom module. Each single-core binary module supports four binary schemes, and the four modules are independent of each other. The third stage is to type each data after binarization into a packet containing the current bin value, the coding type, and the contextual model index. These data are then integrated into the PIPO module and passed into the arithmetic encoding and the context model module as required to achieve a pipeline architecture for the entire entropy encoding module [22].

## 5. Implementation Results

Experiments are conducted to evaluate the performance of the proposed architecture, and the superiority of the proposed CABAC encoder is tested via the HEVC reference software HM-16.7. The proposed CABAC encoder is implemented in Verilog HDL. RTL simulation is performed on 18 sequences in 5 classes. Tests cover All Intra (AI), Low Delay (LD), Low Delay P, and Random Access (RA) configurations and include settings for Quantization Parameters (QPs) 22 and 37.

The CABAC pipeline 1 bin/clock architecture designed in this paper avoids the pipeline stall problem. Table 4 presents the encoding time that can be saved when encoding a video sequence since the approach in this paper avoids the stall of the pipeline architecture caused by successive identical context models. Under general test settings in the AI configuration, the suggested CABAC architecture can save up to 45.66% of the coding time by employing the prediction-based context model prefetching method. Even in the LD, LD_P, and RA settings, the encoding time can have significant reductions. When the QP is low, the encoding time can be reduced by 27.5% on average, and even when the QP is 37, the pipeline architecture stalls can be optimized by 20.95% on average. This is because the context model prefetching architecture proposed in this paper can be adapted to the pipeline architecture to avoid the time consumption caused by the context model update in memory. The time savings differ since low QP values for high-resolution video increase the SEs associated with coding residuals. These SEs provide many bins with the same contextual model for standard coding.

This paper’s architecture follows the anticipated strategy, allowing it to avoid pipeline standstill brought on by context model updates and allocate the number of codes per group of bins through the prefetching module in the parallel architecture, which significantly increases coding efficiency. Our proposed CABAC encoder is implemented in Verilog. RTL simulations are performed using the bin sequences in Table 4 across five different resolution classes, and the QPs are 22 and 37. Table 5 shows the effect of LCMU in the simulation. With the LCMU, the number of delivered bins per clock cycle (BPCC) is slightly reduced to below 4, but the maximum clock frequency is substantially increased. Further using VBBI, the final BPCC is between 4.10 and 4.39 (depending on the configuration). Table 6 shows the probability that the parallel architecture stalls due to untimely model reads caused by the next set of bins having the same contextual model as the previous set, the coding time that can be optimized via the prefetching architecture proposed in this paper.

For the full pipelined architecture CABAC, the gate count is 39.52 K, the maximum operating frequency is 714 MHz, and the maximum throughput is 714 Mbin/s. For the CABAC with the highly compatible parallel architecture in this paper, the overall CABAC throughput, at 513 MHZ, is 2191 Mbin/s. Numerous predictive lookup tables and alternative algorithms are required to raise the frequency and the number of parallel bins, and these efforts have led to higher throughput. Therefore, the throughput rate is also the highest due to the optimization of the hardware design and more advanced processes in this work.

**Figure 12:**
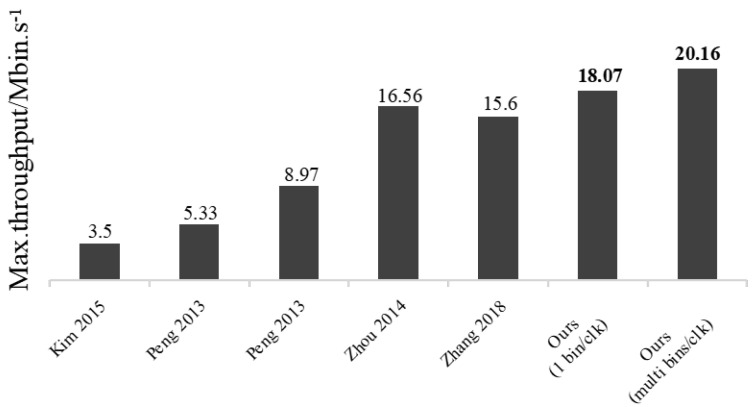
The proposed optimizations improved the hardware efficiency of CABAC when compared to existing papers [12,13,18,29].

Table 7 summarizes the design specifications of our CABAC encoder, compared with the state of the art. Many authors have reported on the CABAC architecture in the past, with their focus on different ASIC technologies. The pipeline architectures of [10,11,12] have similar configurations. Nonetheless, this paper achieves higher throughput rates and smaller circuit areas by targeting critical path optimization for the renormalization part of the *range* update, context model accesses using RAM only, binarization using a single core and more advanced ASIC processes. Ref. [16] parallel architecture designs use the bypass bin splitting technique and merge bypass bins, respectively, to increase the throughput rate. Although their bins per clock cycle are slightly higher than this paper, they pay a high price, such as adding bins splitting/merging modules and PIPOs for storing data such as ranges, etc. The proposed architecture in this research enhances the frequency using the pre-renormalize technique and MCMU, while increasing the throughput by utilizing VBBI, ultimately resulting in improved hardware efficiency, as illustrated in Figure 12. Specifically, the hardware efficiency (Mbins/s per k gates) achieved in this paper is higher than that of other architectures, including both pipeline and parallel architectures; this work achieves 20.16 Mbins/s per k gates. Additionally, the context model prefetching strategy employed in this paper effectively eliminates the time delay that arises due to model updates in memory, effectively mitigating the BAE stagnation problem.

## 6. Summary

The occurrence of consecutive bins in the same context model can cause stalls in the hardware pipeline architecture. To overcome this problem, we propose a prediction-based context model prefetching strategy to alleviate data dependencies by predicting the next bin model, and reduce critical path delays through the MCMU. In addition, we use pre-range update and pre-renormalize technique to reduce the multiplex BAE’s route delay due to the incomplete reliance of the encoding process. Then, we propose the VBBI technique to improve the throughput of BAE in a parallel architecture. Moreover, the data interaction between CABAC modules is optimized. In accordance with the experiments, our architecture eliminates pipeline stalls and saves encoding time, and works better for high resolution and low QP values, which is in line with the need for more high-definition videos as time progresses. Moreover, the throughput is enhanced and the hardware efficiency of the pipeline architecture is maximized. In future study, we will focus on making this work compatible with the multi-channel parallel architecture and Versatile Video Coding (VVC/H.266) hardware design [30].

## Figures and Tables

**Table 1:** All cases with the context model dependencies for the four-way parallel bin.

Bin type	A	B	C	D
Forms used	ST	ST	ST	ST
The result of use	1	2	3S	4
Bin type	A	A	C	D
Forms used	ST	SDT1	ST	ST
The result of use	1	5	3S	4
Bin type	A	A	A	D
Forms used	ST	SDT1	SDT2 + ST	ST
The result of use	1	5	3D	4
Bin type	A	A	A	A
Forms used	ST	SDT1	SDT2 + ST	QT
The result of use	1	5	3D	NG
Bin type	A	B	B	D
Forms used	ST	ST	SDT2	ST
The result of use	1	2	6	4
Bin type	A	B	B	B
Forms used	ST	ST	SDT1	SDT2 + ST
The result of use	1	2	5	3D
Bin type	A	B	C	C
Forms used	ST	ST	ST	SDT2
The result of use	1	2	3	6

**Table 2:** Renormalization times table.

rLPS [7:3]	0	1	2–3	4–7	8–15	16–31
Renormalization times	6	5	4	3	2	1

**Table 3:** Low update with different coding methods.

Coding Method	Input	*Low* Update
Regular	MPS	low≪renorm_count
Regular	LPS	(low+rMPS)≪renorm_count
Bypass	1	low≪1+range
Bypass	0	low≪1

**Table 4:** Percentage of encoding time (%) saved by the CABAC pipeline architecture improving the proposed prediction-based contextual model prefetching strategy under common test conditions.

		All Intra (AI)		Low Delay (LD)		Random Access (RA)		Average	
Class	Sequence	qp = 22	qp = 37	qp = 22	qp = 37	qp = 22	qp = 37	qp = 22	qp = 37
A	PeopleOnStreet	35.50	25.56	30.78	18.47	29.03	18.96	31.28	20.28
	Traffic	30.80	29.76	22.73	20.63	23.66	23.02	24.75	23.30
B	ParkScene	34.86	32.70	25.68	21.23	26.58	23.27	28.01	24.29
	Kimono1	**45.66**	37.45	38.03	28.25	38.29	28.82	39.71	30.39
	BasketballDrive	34.54	25.84	33.05	23.94	34.05	23.94	33.08	23.42
	BQ Terrace	38.38	27.96	32.63	23.29	32.99	24.49	33.97	24.33
	Cactus	31.47	27.70	30.34	21.82	28.14	22.74	29.64	23.38
C	BasketballDrill	22.01	21.19	23.54	17.90	22.03	18.07	22.57	18.70
	BQ Mall	25.16	25.30	23.19	18.24	23.19	19.66	23.55	20.31
	PartyScene	23.30	22.29	21.43	18.40	22.10	18.18	21.95	19.13
	Race Horses	35.38	27.48	29.24	17.95	28.35	19.81	30.56	20.81
D	BasketballPass	26.16	22.20	22.27	17.75	22.00	18.04	23.10	18.92
	Blowing Bubbles	22.80	22.98	19.44	16.71	21.30	18.23	20.68	18.67
	BQSquare	27.08	21.58	18.44	11.96	17.85	15.33	20.36	15.16
	Race Horses	32.46	21.59	21.94	16.72	22.92	17.04	24.81	18.02
E	Kristen And Sara	29.38	24.30	29.93	14.84	28.88	18.63	29.48	18.07
	FourPeople	27.39	26.22	26.85	16.39	26.73	20.78	26.81	19.91
	Johnny	33.03	26.30	30.08	17.08	30.57	20.05	30.76	20.08
Average		**30.85**	26.02	26.64	18.98	26.59	20.50	**27.50**	20.95

**Table 5:** Percentage of coding time (%) saved via the prediction-based context model prefetching strategy proposed by the CABAC parallel architecture improvement.

Sequence	Config.	qp = 22	qp = 27	qp = 32	qp = 37
BasketballDrive	LD	30.39	25.51	22.27	18.52
	RA	30.04	24.46	21.30	18.07
Traffic	LD	22.09	22.36	20.96	18.83
	RA	22.31	23.04	22.27	20.63
PeopleOnStreet	LD	27.84	22.56	18.69	15.86
	RA	25.54	21.19	18.08	15.80
BQTerrace	LD	33.45	27.03	25.49	22.31
	RA	32.80	26.34	23.83	22.89
Kimono	LD	34.55	32.20	29.62	26.33
	RA	33.52	31.12	28.46	25.61
Average	**24.60** *	**29.25**	25.58	23.10	20.48

* Overall average.

**Table 6:** Performance in number of delivered BPCC for H.265/HEVC.

Sequence	Config.	LCMU	LCMU + VBBI
BasketballDrive	LD qp = 22	3.90	4.23
	LD qp = 37	3.96	4.28
	RA qp = 22	3.90	4.26
	RA qp = 37	3.96	4.33
Traffic	LD qp = 22	3.93	4.22
	LD qp = 37	3.96	4.25
	RA qp = 22	3.93	4.29
	RA qp = 37	3.94	4.29
PeopleOnStreet	LD qp = 22	3.90	4.32
	LD qp = 37	3.97	4.37
	RA qp = 22	3.91	4.38
	RA qp = 37	3.97	4.42
BQTerrace	LD qp = 22	3.86	4.10
	LD qp = 37	3.93	4.23
	RA qp = 22	3.87	4.15
	RA qp = 37	3.93	4.27
Kimono	LD qp = 22	3.86	4.26
	LD qp = 37	3.91	4.21
	RA qp = 22	3.87	4.34
	RA qp = 37	3.91	4.28
Average		3.92	**4.27**

**Table 7:** Specification and comparison with prior arts.

Design	Kim [12]	Peng [13]	Ding [14]	Zhou [18]	Zhang [29]	This Work	
Process/nm	IDEC 180	TSMC 130	Kintex-7	TSMC 90	TSMC 90	TSMC 65	
gate count/K	45.089	48.94	-	110.9	54.5	39.52	108.7
Max.clock frequency/MHZ	158	357	120	420	720	714	513
bins/clock	1	1.18	3.59	3.29 (4.37) *	-	1	4.27
Max·throughput/Mbin·s${}^{-1}$	158	261–439	431	1382 (1836 ) *	850	714	**2191**
Mbin·s${}^{-1}$/gate count	3.5	5.33–8.97	-	12.48 (16.56 ) *	15.6	**18.07**	**20.16**

* The actual results, the ones in parentheses are the occasional optimal results.

## Data Availability

Data sharing is not applicable to this article.

## Abbreviations

The following abbreviations are used in this manuscript:

IoT	Internet of Things
CABAC	Context Adaptive Binary Arithmetic Coding
HEVC	High Efficiency Video Coding
MCMU	Multi-Result Context Model Update
VBBI	Variable Bypass Bin Incorporation
QP	Quantization Parameter
UHDTV	Ultra High Definition Television
JCT-VC	The Joint Collaborative Team on Video Coding
CTUs	Coding Tree Units
CAVLC	Context-based Adaptive Variable Length Coding
RDO	Rate Distortion 0ptimization
AVC	Advanced Video Coding
SEs	Syntax Elements
BEE	Bypass Encoding Engines
MBBS	Multiple Bypass Bins Scheme
BAE	Binary Arithmetic Encoding
RTL	Register Transfer Level
MRSET	Multiple Residual Syntax Element Treatment
PIPO	Parallel-In-Parallel-Out
FL	Fix Length
TU	Truncated Unary
TR	Truncated Rice
MPS	More Probable Symbol
LPS	Less Probable Symbol
SRAM	Static Randomaccess Memory
SDT	State Dual-Transition
PISO	Parallel In Serial Out
ST	State Transition
BPBS	Bypass Bin Splitting
FIFO	First-Input-First-Output
WP	Write Pointer
RP	Read Pointer
AI	All Intra
LD	Low Delay
RA	Random Access
BPCC	Bins Per Clock Cycle
VVC	Versatile Video Coding
